# Vaccinate fast but leave no one behind: a call to action for COVID-19 vaccination in Spain

**DOI:** 10.1038/s43856-021-00014-2

**Published:** 2021-07-07

**Authors:** Jeffrey V. Lazarus, Quique Bassat, Javier Crespo, Gonzalo Fanjul, Alberto L. Garcia-Basteiro, Marcos López Hoyos, Carlos Mateos, José Muñoz Gutierrez, Denise Naniche, Miquel Oliu-Barton, Kenneth H. Rabin, Rafael Vilasanjuan, Sonia Villapol, Jose M. Martin-Moreno

**Affiliations:** 1 Barcelona Institute for Global Health (ISGlobal), Hospital Clínic de Barcelona, University of Barcelona, Barcelona, Spain; 2 Centro de Investigaçäo em Saúde de Manhiça (CISM), Maputo, Mozambique; 3 Institución Catalana de Investigación y Estudios Avanzados (ICREA), Barcelona, Spain; 4 Hospital Sant Joan de Déu, University of Barcelona, Barcelona, Spain; 5 Consorcio de Investigación Biomédica en Red de Epidemiología y Salud Pública (CIBERESP), Madrid, Spain; 6 Sociedad Española de Patología Digestiva (SEPD), Madrid, Spain; 7 Hospital Universitario de Marqués de Valdecilla, Santander, Cantabria Spain; 8 Sociedad Española de Inmunología (SEI), Barcelona, Spain; 9 Instituto de investigación sanitaria Valdecilla (IDIVAL), Santander, Cantabria Spain; 10 Instituto #SaludsinBulos, Madrid, Spain; 11 Academia Internacional de Estudios Superiores (AIES), Barcelona, Spain; 12 Hospital Clínic de Barcelona, University of Barcelona, Barcelona, Spain; 13 Université Paris-Dauphine – PSL, Paris, France; 14 ESADE Centre for Economic Policy, Madrid, Spain; 15 CUNY Graduate School of Public Health & Health Policy, New York, United States; 16 Houston Methodist Research Institute, Houston, Texas United States; 17 Weill Cornell Medical College, New York, United States; 18 Department of Preventive Medicine and Public Health, Medical School and INCLIVA-Clinical Hospital, University of Valencia, Valencia, Spain

**Keywords:** Public health, Viral infection

## Abstract

Lazarus et al. outline the barriers slowing down the COVID-19 vaccination campaign in Spain. They issue a call to action for all stakeholders to improve access to vaccines, with a particular emphasis on reaching marginalised populations.

## Teaser

During the first five months of 2021, Spain’s COVID-19 vaccination campaign progressed slowly and failed to reach marginalised populations. Here, we discuss how, despite recent improvements, it remains important to further engage key stakeholders to ensure nobody is left behind.

Vaccines prevent disease and premature deaths, strengthen and build trust in health systems, and make communities more productive^1^. They represent one of the most cost-effective ways to reduce morbidity and mortality, preventing approximately 2–3 million deaths annually worldwide^2^.

Addressing the needs of marginalised groups will require greater engagement and innovation in public health practice

Today, factors such as a lack of confidence in vaccines^3^, complacency that is fuelled, in part by individualism, and the success of infectious disease control in many parts of the world, disinformation, and limited access to vaccines have led to serious declines in immunization rates^4^. The World Health Organization (WHO) declared in 2019 that vaccine hesitancy was one of the ten greatest global health threats, alongside antimicrobial resistance, human immunodeficiency virus (HIV), and climate change^2^. Vaccine hesitancy can have grave effects, such as the global increase in the incidence of vaccine-preventable diseases such as measles, with recent outbreaks occurring in middle- and high-income countries^5^. Vaccine confidence varies significantly around the world and is dangerously low and falling in some countries. Hesitancy occurs in low-, middle-, and high-income countries alike, and may be found among people in all socioeconomic, religious, and ethnic groups^6^. The trend of increased hesitancy and lack of confidence in vaccines is also associated with a broader erosion of public trust in scientific and governmental efforts to maintain public health^7^. In the context of the COVID-19 pandemic, this erosion has been exacerbated by the speed and intensity of information exchange, in news coverage, and social media. The public’s capacity to distinguish reliable, evidence-based information from falsehoods has been compromised, a phenomenon described by the WHO as an infodemic^8^.

During a pandemic, vaccines must reach the greatest number of people in any given country but, most importantly, globally. Safe and effective vaccines against COVID-19 were developed very quickly once the genome of the virus was sequenced. From December 2020 to early June 2021, more than 2 billion people have been vaccinated against COVID-19, but with great inequity among and within countries. Mechanisms to foster a sense of collective responsibility are needed to ensure affordability and sustainable financing of COVID-19 vaccines in low- and middle-income countries, which are home to about 85% of the global population and often lack the resources to purchase, stock, and distribute adequate quantities of vaccines. This is why we unconditionally support the concept of vaccine internationalism, favouring an equitable and coordinated global vaccination approach as stated by WHO’s Vaccine Equity Declaration, the COVID-19 Vaccines Global Access (COVAX) initiative, and the COVID-19 Vaccine Equity Project. However, even in high-income countries, like Spain, it is vital to ensure access to COVID-19 vaccines for marginalised populations, such as migrants, the homeless, people who use drugs, certain ethnic minorities, and the very poor, as they are the ones with major difficulties in accessing the health system^4^. Not only is this the right thing to do from a humanitarian perspective, but ultimately herd immunity is needed to create a firewall against the transmission of the disease and the generation of new variants, and it will be less successful if parts of the population are left largely unvaccinated. In this overall context, the positive public health benefits of newly available effective COVID-19 vaccines, including a single-dose vaccine, have not been realised in Spain.

Everyone, from political leaders to the general population, should understand that we will not be safe from COVID-19 until everyone is protected from this highly transmissible and life-threatening disease. Spain has already been disproportionately affected: as of June 7, 2021, Spain officially accounts for ~2.1% of all reported COVID-19 fatalities, despite representing only 0.6% of the world’s population^9^. Evidence from the first 12 months of the pandemic shows that health, the economy, and civil liberties are not in conflict but go hand in hand^10^. Here, we discuss the specific challenges for Spain and outline the actions needed to vaccinate the vast majority of the population, including marginalised groups.

## Addressing specific vaccination challenges in Spain

In Spain, healthcare services are decentralised and health competencies are delegated to each of its 17 regions, known as autonomous communities. The devolution of power to the level of a specific region was a good faith attempt to make the work of each of the regional health services, which together constitute the national health system, more agile and responsive to local needs. However, inequalities, for example in the number of healthcare professionals employed, have increased in this system since the economic crisis a decade ago, and could be an added factor contributing to variation in regional COVID-19 vaccination rates. For this reason, we are now issuing a call to action for COVID-19 vaccination in Spain.

Spain’s goal of vaccinating 70% of the adult population by the end of the summer of 2021, in line with the objectives set by the European Commission^11^, may be achieved in spite of the slow pace of vaccination up to early June 2021, yet there is no clear vision beyond the summer. Recent uncertainty surrounding rare adverse events associated with the AstraZeneca and Johnson & Johnson vaccines and the way these have been factored into government risk-benefit analyses have reinforced both supply constraints and vaccine hesitancy and represent an additional barrier to raising national vaccination rates and reaching those that are still most in need. Importantly, the recurrent reporting of negative news (for instance the vaccines’ rare adverse events) in contrast to the publishing of success stories (such as the vaccines’ proven capacity to save lives) further impacts people’s perceptions of the risks and benefits of current policy recommendations. This has been compounded by the confusion generated by the new option in Spain of combining different COVID-19 vaccines based on limited evidence.

Even when current difficulties are eased, we can anticipate continued problems in meeting the needs of marginalised populations. Spanish vaccination efforts have been supported by scientific societies, such as the ten points put forward by the Spanish Vaccinology Association in December 2020^12^, which are designed to promote COVID-19 vaccine coverage through expanded public dialogue and education that will enable individuals, communities, and government leaders to better understand the role of vaccination, make more informed choices about its use, and sustain investment in expanded access to immunization. In April 2021, 82 Spanish scientific societies called for the government to continue to work towards herd immunity^13^. Their 11 action points include combatting the infodemic contributing to vaccine hesitancy, and highlight the failure to address the critical challenge of clear communication to the public as a shortcoming of governments in Spain^7,14^. At the end of April 2021, 18 scientific societies again offered to assist the government with their vaccination programme^15^. None of these declarations address or even mention marginalised populations, an issue that is likewise absent in the governments’ COVID-19 vaccination programmes, which are focused on vaccinating by age and vaccinating healthcare professionals.

Addressing the needs of marginalised groups will require greater engagement and innovation in public health practice. Delivery models such as offering new vaccination services like mobile units or immediately allowing vaccination to all ages among marginalised populations must be considered. Additional human resources may be needed to administer vaccines more quickly, especially if it means going outside of clinical settings to reach marginalised populations in their communities. To meet the short-term needs for trained health workers, medical and nursing school students and veterinarians could be authorised to vaccinate. Community pharmacies should also be empowered to reinforce recommendations regarding prevention and protection. In the next section, we pay special attention to those who are often left behind in the current and future priorities for COVID-19 vaccination in Spain.

## Priorities for COVID-19 immunization action in Spain

In Box 1 we make specific recommendations for improving access to the COVID-19 vaccine with an emphasis on reaching marginalised populations. National and regional governments, supported by key stakeholders, must lead the vaccination effort to bring the COVID-19 pandemic under control. Governments must, for example, provide reliable information endorsed by trustworthy sources from throughout society, including health professionals and their scientific societies, patients and other non-governmental organisations, faith-based and community leaders, and other well-known figures. In particular, European governments should strongly endorse and disseminate the European Medicines Agency (EMA) recommendations to the public and encourage the European Union (EU) to act as a bloc based on the best available scientific evidence, especially with regard to vaccines. Such consistent messaging across Europe will help re-establish societal trust in vaccination as a foundation of public health action.

Many marginalised populations are best reached by non-governmental and community-based organisations. These organisations may need additional support and capacity from the government, media, and researchers to support the full realisation of COVID-19 vaccination programmes. Nevertheless, primary care doctors, pharmacists, and nurses can also play a central role in supporting and informing this population. As the gateway to the health system across Spain, these professionals must be better supported during the pandemic so that together with other key stakeholders they can improve vaccination rates and reach all marginalised populations.

Box 1. Recommendations to improve access to the COVID-19 vaccine in Spain ***For governments and policy-makers***

- Disseminate widely accurate COVID-19 immunization information targeted to the right audience. Information should be communicated in plain language, with images as needed, via direct mailings to all homes, via mass and social media, and to marginalised populations including at religious services, homeless shelters, and community centres.
- Promulgate regulations that encourage vaccination (e.g. fewer social and travel restrictions for vaccinated people). Consider adopting a national health pass that includes vaccination, as in other European countries, to facilitate travel and social activities such as attending selected public events.
- Strengthen and empower the independent scientific advisory panel to regularly advise on response measures across the entire vaccine implementation process. In addition to medical scientists and experts in public health, this panel should be multidisciplinary, and include economists, experts working with marginalised populations, such as sociologists, political scientists, and anthropologists.
- Spain should share doses with COVAX in parallel with the national vaccine rollout, especially if there is a decision not to use or limit the use of a safe and effective vaccine. Children are at reduced risk of severe COVID-19, and should thus be deprioritised in the vaccination effort in order to support efforts abroad.
- Given that several variants of concern have already been identified to date, virological surveillance of variants based on whole-genome sequencing should be reinforced to at least 5% - and preferably higher - of all positive specimens, in line with European Commission guidance – and special attention should be given to returning travellers.

***For advocacy groups***

- Support and facilitate access to immunization for all marginalised populations.
- Involve community leaders, including current, and former COVID-19 patients, to disseminate accurate information on vaccines.
- Leverage the care pathways established to diagnose some pathologies such as HIV, and the micro-elimination circuits of hepatitis C, to facilitate the vaccination of highly marginalised populations.

***For healthcare professionals***

- Commit to listen non-judgmentally to people’s concerns about vaccination and play a proactive role in helping to overcome barriers to immunization.
- Contribute to health literacy by correcting misleading information and sharing evidence-based information. This includes counselling patients about vaccine safety.
- Improve coordination and communication between health and social services to support COVID-19 data collection and dissemination.
- Contribute regular and comprehensive service-delivery assessments to monitor and overcome ongoing barriers for marginalised populations to access COVID-19 immunization services.
***For researchers and scientific communities***
- Conduct qualitative and quantitative research on vaccination and on willingness to be vaccinated, and contribute to evidence-based communication programmes to strengthen vaccine literacy, especially among marginalised populations.
- For informatics experts, collaborate with health professionals to build tools to detect and respond to false information about vaccination.
- Create multidisciplinary pandemic control expert groups within and across scientific societies. Such groups should also have an international component, and include representatives from marginalised populations.
***For media and social media professionals***
- Provide evidence-based, easy-to-understand information about the importance of everyone getting vaccinated, and actively combat the widespread disinformation, and misinformation about vaccines and the pandemic.
- Continue to interview and cite vetted leading experts to raise awareness about the COVID-19 vaccines.

## References

[1] World Health Organization. Global vaccine action plan 2011-2020. https://www.who.int/publications/i/item/global-vaccine-action-plan-2011-2020 (2013).

[2] World Health Organization. Ten threats to global health in 2019. https://www.who.int/news-room/spotlight/ten-threats-to-global-health-in-2019 (2019).

[3] Sallam M (2021). COVID-19 vaccine hesitancy worldwide: a concise systematic review of vaccine acceptance rates. Vaccines.

[4] Wouters OJ (2021). Challenges in ensuring global access to COVID-19 vaccines: production, affordability, allocation, and deployment. Lancet..

[5] Hotez PJ, Nuzhath T, Colwell B (2020). Combating vaccine hesitancy and other 21^st^ century social determinants in the global fight against measles. Curr. Opin. Virol..

[6] Lazarus JV (2020). Hesitant or not? The association of age, gender, and education with potential acceptance of a COVID-19 vaccine: a country-level analysis. J. Health Commun..

[7] Scholz, N. Covid-19 vaccination campaigns: the public dimension. https://www.europarl.europa.eu/RegData/etudes/BRIE/2021/679063/EPRS_BRI(2021)679063_EN.pdf (2021).

[8] World Health Organization. Infodemic. https://www.who.int/health-topics/infodemic#tab=tab_1 (2021).

[9] Worldometer. Reported cases and deaths by country or territory. https://www.worldometers.info/coronavirus/#countries (2021).

[10] Oliu Barton M (2021). SARS-CoV-2 elimination, not mitigation, creates best outcomes for health, the economy, and civil liberties. Lancet.

[11] European Commission. EU vaccines strategy. https://ec.europa.eu/info/live-work-travel-eu/coronavirus-response/public-health/eu-vaccines-strategy_en (2021).

[12] Asociación Española de Vacunología. Decálogo a favor de las vacunas frente a la COVID-19. https://www.vacunas.org/decalogo-a-favor-de-las-vacunas-frente-a-la-covid-19/ (2020).

[13] 2° Congreso Nacional Multidisciplinar Covid-19 de las Sociedades Científicas de España. Manifiesto: no paren la vacunación y pónganse de acuerdo en las medidas para el control de la pandemia. https://2congresocovid.es/2congresocovid (2021).

[14] White, T. M. et al. COVID-SCORE Spain: public perceptions of key government COVID-19 control measures. *Eur. J. Public Health*10.1093/eurpub/ckab066 (2021). 10.1093/eurpub/ckab066 PMC8083190 33872348

[15] Sociedad Española de Medicina de Urgencias y Emergencias. Comunicado de 18 Sociedades Científicas sobre las vacunas para la Covid-19. https://www.semes.org/comunicado-de-18-sociedades-cientificas-sobre-las-vacunas-para-la-covid-19/ (2021).

